# Notes and News

**Published:** 1905-07-01

**Authors:** 


### NOTES AND NEWS.

THE annual meeting of the Association for the Oral Instruction of the Deaf and Dumb will be held at 3.30 on July 5th at the Portman Rooms.

MR. T. DYER EDWARDES, J.P., has been elected chairman of the council of the Chelsea Hospital for Women, and Sir Frederic W. Fryer, K.C.S.I., has been elected vice-chairman.

THROUGH the will of the late Mr. Wyndham Francis Cook, of Messrs. Cook and Co. of St. Paul's Churchyard, the Hospital Sunday Fund has received £12,000. The Lord Mayor has also received £1,000 each from Mr. Charles Morrison and Mr. William Herring.

WE regret to announce the death of Inspector-General of Hospitals and Fleets, Alexander Turnbull. His last appointment was at Haslar Hospital, where aseptic operating rooms are enumerated amongst the many reforms effected by him.

A MEETING under the presidency of the Duke of Northumberland will be held at the Middlesex Hospital on the 30th inst. at five o'clock, to consider the question of the support of medical schools out of hospital funds, raised by King Edward's Hospital Fund.

THE death of the distinguished surgeon, Professor von Mikulecz, has occurred at Breslau. He delivered the Cavendish Lecture in 1904. He collaborated with Professor von Bergmann and Professor von Bruns in a "System of Practical Surgery," which has been translated into English by Professor Bull of New York.

THE Royal Commission on the Care of the Feeble-Minded are continuing their sittings. Mr. Arthur Acland Allen suggested that suitable cases should be sent to day centres up to the age of 18; that unsuitable cases should be drafted to residential homes, and that it should be compulsory on some authority to make provision for the care and instruction of imbeciles.

THE Hospital Sunday Fund announces a collection of £25,000 up to the present date. This includes Mr. Cook's legacy and the various generous gifts which have been received recently.

WE regret to record the death of Dr. James MacNab Cunningham, M.D., LL.D., C.S.I., honorary surgeon to the King. He was attached to the Indian Medical Department, in connection with which he had a long and useful career.

THE present framing of the Child Courts Bill makes no provision for the presence of the Press in the court. The Humanitarian League protests against this exclusion on the ground that publicity is essential as a safeguard against unjust sentences.

WE learn that Captain Davenport, aide-de-camp to Lord Milner, has been appointed secretary-superintendent at St. George's Hospital. We understand that Captain Davenport has not much acquaintance with hospitals, but as Mr. C. L. Todd, the able secretary-superintendent, will remain in office, probably until the end of the year, he will be able to avail himself of the large amount of knowledge which Mr. Todd has acquired during 44 years spent in the service of the hospital.

THE Guy Medal in silver has been awarded to Mr. R. Henry Rew for his work in connection with the preparation of the Reports of the Special Committee appointed by the Royal Statistical Society to investigate the Production and Consumption of Meat and Milk in the United Kingdom, and for his paper entitled "Observations on the Production of Meat and Dairy Products." The tenth session of the International Statistical Institute will be held in London at the Imperial Institute, South Kensington, from July 31st to August 5th next. The Honorary President of the Society, H.R.H. the Prince of Wales, has consented to act as Honorary President of the Congress, and to preside at the opening meeting on Monday, July 31st, at 11 A.M.

UNDER the heading "The Latest Scandal" a Maltese paper publishes a very sensational article on the appointment of an English medical (military) man on the board of the Lunatic Asylum. This appointment has been made a grievance by the Maltese members of the board, and the journal referred to states that "the appointment was made for the sole and unmistakable object of controlling and checking the work of the Maltese medical men" in regard to English patients. On the face of it, the whole matter appears to us to be a storm in a teacup, which could easily have been avoided by a little tact on the one hand, with reasonableness and less susceptibility to take offence on the other,—unless, of course, the action of the English Government has been welcomed to aid imaginary grievances and create an ill-feeling, which the *Malta e Sue Dipendenza* professes a desire to avoid. The title of the article is not very happy in the cause of peace. The British Medical Association and the Government have been appealed to, so it is to be hoped that angry feelings will be soothed and that a reasonable spirit will prevail.

